# Research on Time Series Monitoring of Surface Deformation in Tongliao Urban Area Based on SBAS-PS-DS-InSAR

**DOI:** 10.3390/s24041169

**Published:** 2024-02-10

**Authors:** Yuejuan Chen, Cong Ding, Pingping Huang, Bo Yin, Weixian Tan, Yaolong Qi, Wei Xu, Siai Du

**Affiliations:** 1 College of Information Engineering, Inner Mongolia University of Technology, Hohhot 010080, China; chen_yj@imut.edu.cn (Y.C.); 20211100106@imut.edu.cn (C.D.); hwangpp@imut.edu.cn (P.H.); wxtan@imut.edu.cn (W.T.); qiyaolong@imut.edu.cn (Y.Q.); xuwei1983@imut.edu.cn (W.X.); 20211800106@imut.edu.cn (S.D.); 2 Inner Mongolia Key Laboratory of Radar Technology and Application, Hohhot 010051, China; 3 College of Resource and Environmental Engineering, Inner Mongolia University of Technology, Hohhot 010080, China

**Keywords:** InSAR, urban areas, surface deformation monitoring, ground control points

## Abstract

As urban economies flourish and populations become increasingly concentrated, urban surface deformation has emerged as a critical factor in city planning that cannot be overlooked. Surface deformation in urban areas can lead to deformations in structural supports of infrastructure such as road bases and bridges, thereby posing a serious threat to public safety and creating significant safety hazards. Consequently, research focusing on the monitoring of urban surface deformation holds paramount importance. Interferometric synthetic aperture radar (InSAR), as an important means of earth observation, has all-day, wide-range, high-precision, etc., characteristics and is widely used in the field of surface deformation monitoring. However, traditional solitary InSAR techniques are limited in their application scenarios and computational characteristics. Additionally, the manual selection of ground control points (GCPs) is fraught with errors and uncertainties. Permanent scatterers (PS) can maintain high interferometric coherence in man-made building areas, and distributed scatterers (DS) usually show moderate coherence in areas with short vegetation; the combination of DS and PS solves the problem of manually selecting GCPs during track re-flattening and regrading, which affects the monitoring results. In this paper, 45 Sentinel-1B data from 16 February 2019 to 14 December 2021 are used as the data source in the urban area of Horqin District, Tongliao City, Inner Mongolia Autonomous Region, for example. A four-threshold (coherence coefficient threshold, FaSHPS adaptive threshold, amplitude divergence index threshold, and deformation velocity interval) GCPs point screening method for PS-DS, as well as a Small Baseline Subset-Permanent Scatterers-Distributed Scatterers-Interferometric Synthetic Aperture Radar (SBAS-PS-DS-InSAR) method for selecting PS and DS points as ground control points for orbit refinement and re-flattening, are proposed. The surface deformation results obtained using the Small Baseline Subset Interferometric Synthetic Aperture Radar (SBAS-InSAR) and the SBAS-PS-DS-InSAR proposed in this paper were comparatively analysed and verified. The maximum cumulative line-of-sight settlements were −90.78 mm and −83.68 mm, and the maximum cumulative uplifts are 74.94 mm and 97.56 mm, respectively; the maximum annual average line-of-sight settlements are −35.38 mm/y and −30.38 mm/y, and the maximum annual average uplifts are 25.27 mm/y and 27.92 mm/y. The results were evaluated and analysed in terms of correlation, mean absolute error (MAE), and root mean square error (RMSE). The deformation results of the two InSAR methods were evaluated and analysed in terms of correlation, MAE, and RMSE. The errors show that the Pearson correlation coefficients between the vertical settlement results obtained using the SBAS-PS-DS-InSAR method and the GPS monitoring results were closer to 1. The maximum MAE and RMSE were 13.7625 mm and 14.8004 mm, respectively, which are within the acceptable range; this confirms that the monitoring results of the SBAS-PS-DS-InSAR method were better than those of the original SBAS-InSAR method. SBAS-InSAR method, which is valid and reliable. The results show that the surface deformation results obtained using the SBAS-InSAR, SBAS-PS-DS-InSAR, and GPS methods have basically the same settlement locations, extents, distributions, and temporal and spatial settlement patterns. The deformation results obtained using these two InSAR methods correlate well with the GPS monitoring results, and the MAE and RMSE are within acceptable limits. By comparing the deformation information obtained using multiple methods, the surface deformation in urban areas can be better monitored and analysed, and it can also provide scientific references for urban municipal planning and disaster warning.

## 1. Introduction

Surface deformation, as the most common factor triggering geological disasters, has a long duration and a wide range of influence, posing a major threat to the safety of urban infrastructure [1,2,3]. Ground subsidence will damage the natural environment for human life [4], even endangering people’s property and life safety [5], and restrict the comprehensive, coordinated, and sustainable economic and social development of subsidence areas [6]. At the present stage, there are three main techniques for monitoring surface deformation, which are traditional measurement technology, GPS technology, and synthetic aperture radar (SAR) interference technology. However, traditional measurement techniques and GPS technology have lower operational efficiencies, which are insufficient for monitoring large-scale land subsidence [7]. SAR is a kind of radar that can still image with high resolution even under harsh environmental and climatic conditions [8,9]. InSAR technology has the advantages of all-weather, round-the-clock, high-resolution, wide-range monitoring that is low-cost, high-precision, etc., and has been greatly developed in the related applications of surface deformation monitoring [7,10,11].

Differential Interferometric Synthetic Aperture Radar (D-InSAR) is a technique that uses the phase difference of 2- or multi-scene SAR images in combination with external topographic data to obtain the small deformation of the ground surface, but it is easily affected by time and space. However, it is susceptible to temporal as well as spatial incoherence [12,13]. In order to solve this problem in the early 21st century, the Permanent Scatterer Interferometric Synthetic Aperture (PS-InSAR) technique was created, which selects the points with high coherence and stability as the PS point targets, and then analyses the phase characteristics of these target points and separates the corresponding atmospheric phases to obtain accurate ground deformation information [14,15,16,17]. Subsequently, the SBAS-InSAR [18,19] technique was proposed, which employs small baseline combinations for measurements and uses the singular value decomposition (SVD) method to compute multiple small baseline combinations for atmospheric phase removal and deformation inversion in order to efficiently obtain surface deformation information on a time series. After more than a decade of development and application, currently, these two time-series InSAR techniques are commonly used in large-scale surface deformation monitoring [20,21,22,23,24]. Since the density of PS points in non-urban areas is generally less than 10 km^2^, which is much smaller than that in urban areas, it is difficult for PS-InSAR to obtain spatially continuous surface deformation information in areas with few artificial structures. In order to solve this problem, Ferretti proposed the Squee SAR [25] technique, which combines PS and DS to solve this problem and can obtain high densities in non-urban areas without affecting the densities of urban points. This method is able to increase the spatial density of measurement points over areas characterised by DS while preserving the high quality of information obtained using the PS technique on deterministic targets, i.e., spatially averaging the data over statistically homogeneous areas, thus improving the signal-to-noise ratio (SNR) [25].

For surface deformation, many researchers and scholars choose to use InSAR technology to monitor surface deformation. In 2019, Zhong Yahui [26] used the SBAS-InSAR method to invert the surface deformation of the high-resolution radar imagery data in the Changzhou area and obtained annual average subsidence rate and time-series surface cumulative deformation maps in the Changzhou area in the time period. They also found that the results of Changzhou City showed the characteristics of “Basically stable in the whole area, and severe local subsidence in Wujin area”, and analysed the causes of this phenomenon. In 2019, Wang Yimei [27] et al. used the improved SBAS-InSAR technique to study the ground subsidence in Zhengzhou City and obtained its spatial distribution characteristics, then explored the causes of subsidence.

In order to verify the accuracy of the InSAR technique, the experimental results were compared and verified in combination with other surface deformation monitoring means or geological data of the study area. In 2018, Wei Xuemei [28] conducted a subsidence monitoring study in key areas of Hefei City using the small baseline set technique, then analysed and compared subsidence in Hefei City in combination with the measured data as well as groundwater data. Based on SBAS-InSAR technology, Zhou Lu [29] and others studied the spatial and temporal characteristics of surface deformation in central Wuhan, then compared them with 32 local level points to verify the accuracy and reliability of the monitoring results. In 2022, Wang Lei [30] and others processed the image data of the covered study area based on SBAS-InSAR technology, obtained the annual subsidence rate and temporal sequence of deformation in the study area information, and compared the data with PS-InSAR results for verification. Finally, they further analysed the causes of subsidence in the study area. A comparison of SBAS-InSAR and PS-InSAR results showed that the two time-series analysis methods can obtain consistent surface deformation trends in large-scale and continuous slow urban surface deformation monitoring, but SBAS-InSAR is more advantageous in terms of monitoring the details of surface deformation, and the deformation results monitored by the SBAS-InSAR technique are more reliable.

Based on the traditional InSAR, it was improved and able to obtain more accurate deformation results. In 2020, Wei Lianhuan [31] improved the small baseline set technique, proposed a slope displacement solution method based on topographic features, and studied the Dagushan iron ore mine of Ansteel Group as a research object to obtain its spatial distribution characteristics, then combined it with measured data as well as the precipitation data of the mine area until the reliability of the method was verified. In 2022, Yang Wang [32] et al. determined the surface of the Jinchuan mine area from 2018 to 2020 based on Sentinel-1A data from three orbits (ascending orbit 128, descending orbit 33 and 135) using a small base set interferometric radar (SBAS-InSAR) and the least squares iterative method, combining the a priori 3D component deformation rates and time-series deformation variables. They validated the vertically oriented cumulative deformation values using levelled measured data. The selection of GCPs for orbit refinement and re-flattening is an extremely important step for deformation inversion in the InSAR processing flow [33]. Many researchers and scholars have taken a keen interest in this area to improve and upgrade the ground control point screening [34,35,36].

However, traditional InSAR methods for monitoring deformation in urban areas have their own advantages and shortcomings, which are also related to the coherence of the study area. Moreover, when selecting GCPs, it is easy to produce large errors, which affect the accurate inversion of the subsequent deformation results. Aiming at the above problems, this paper combines the characteristics of surface deformation in urban areas and adopts appropriate InSAR processing methods in order to obtain more accurate surface deformation monitoring results. Taking the urban area of Horqin District, Tongliao City, Inner Mongolia Autonomous Region as an example, 45 Sentinel-1B data from 16 February 2019 to 14 December 2021 are used as the data source. A SBAS-PS-DS-InSAR method combining the PS-DS (coherence coefficient threshold, FaSHPS adaptive threshold, amplitude discrepancy index threshold, and conformal velocity interval) four-thresholding method is proposed to select the PS points and DS points as the GCPs for orbit refinement and re-flattening. The surface deformation results obtained using the SBAS-InSAR and the SBAS-PS-DS-InSAR techniques proposed in this paper are comparatively analysed and validated. The deformation results monitored by these two InSAR methods are evaluated and analysed in terms of three indicators: Pearson correlation coefficient, MAE, and RMSE. The use of this method facilitates the real-time understanding of important information such as the risk of geological hazards, the status of groundwater resources, crustal movement, and tectonic evolution in the study area. These data are of great significance for scientific and rational municipal planning, disaster prevention, geological research, etc., and will help to improve the sustainable development of the region.

## 2. Study Area and Methods

### 2.1. Overview of the Study Area

**Figure 1:**
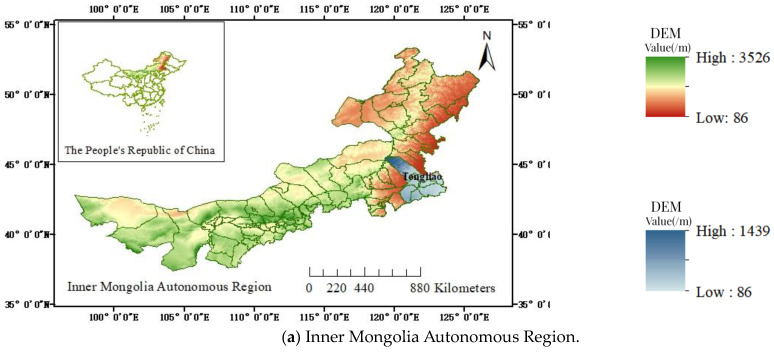

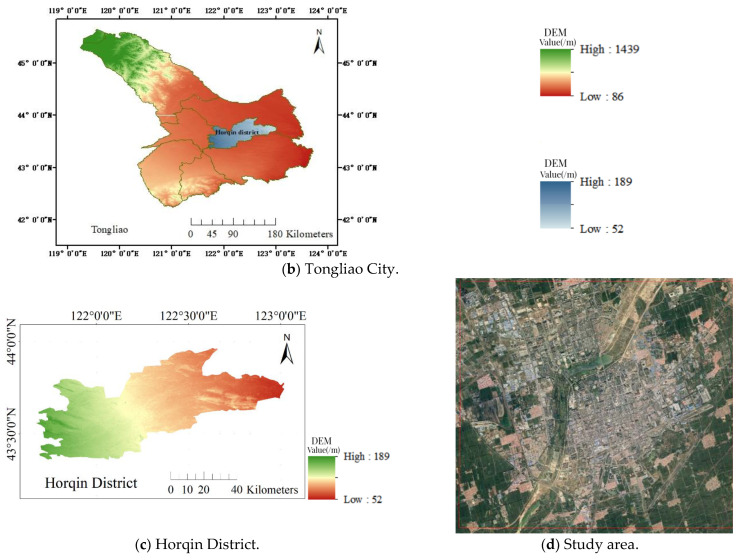
Geographic location and extent of the study area: (**a**) Inner Mongolia Autonomous Region, (**b**) Tongliao City, (**c**) Horqin District, (**d**) study area.

Tongliao City is located in the eastern part of the Inner Mongolia Autonomous Region, on the slope of the Mongolian Plateau descending to the Liaohe Plain, with the general contour of the landscape being high in the south and north and low and flat in the centre. The exact location is shown in Figure 1a. Horqin District is the political, economic, cultural, and transportation centre of Tongliao City, with four distinct seasons; abundant light, rain, and heat at the same time; and moderate temperature. The average annual temperature is 6.1 °C, there are 3113 h of sunshine, the average annual precipitation is 385.1 mm, the average annual frost-free period is 150 days, and the average annual wind speed is 3.6 m/s. Horqin District is located in the southwest area of the Songliao Basin, in the second level of tectonic unit of the Kailu Depression Basin, and the frame of the Ma spit uplifts contact parts. The West Liao River, Qing River, and Hong River flow from west to east through the whole territory, with a total length of 338.4 km. In recent times, due to the flooding of the West Liao River, Qing River, and Hong River, the upper and middle reaches of the alluvial sediments have been deposited into grasslands, with a deposition of a layer of pebbles, fine sand, medium sand, and clay more than 200 metres thick, which is shown in Figure 1b,c. The study area is located in the urban area of Horqin District, Tongliao City, as shown in Figure 1d.

### 2.2. Data

In this paper, the single-look complex (SLC) SAR image data of the descending Sentinel-1B radar satellite were acquired for the 45 views from 16 February 2019 to 14 December 2021 in Horqin District, Tongliao City, Inner Mongolia Autonomous Region. The angle of incidence was 38.96°, and the azimuth was −1.66°. The main parameters of the Sentinel-1B data used in this study are shown in Table 1 and Table 2. The SRTM DEM data provided by NASA, with a ground resolution of 30 m, were used, and the POD precision orbit ephemeris data were provided by ESA. 

In this paper, the VH polarisation is used mainly due to the fact that its radar waves are transmitted vertically and received horizontally, which is suitable for some special features and monitoring needs. This type of polarisation can be used to detect buildings, urban areas, roads, etc., with scattering characteristics [37]. VH polarisation is able to extract more homogeneous points compared to VV polarisation and has higher principal values than VV polarisation [38]. 

### 2.3. Time Series InSAR Methods

#### 2.3.1. PS-InSAR Method

The PS-InSAR technique is mainly used to invert surface deformation by taking advantage of the fact that permanent scatterers are less affected by spatio-temporal incoherence. The basic idea is to select multiple SAR images of the same study area in a certain time period and detect the targets that preserve stable coherence of radar wave backscattering in the imaging area under the long spatial and temporal baselines of multiple SAR images, i.e., PS, which are mainly distributed in man-made buildings, exposed rocks, etc. These feature targets can cause the radar echo signals to preserve a more stable state for a long period of time. The PS network is then solved by modelling and analysing the phase time series of these PS points, so as to separate the information regarding deformation and atmospheric delay and finally obtain the surface deformation information. PS has strong coherence, which reduces the effect of spatial and temporal incoherence on the quality of the interferometric phase, and the PS-InSAR technique can determine the millimetre-level accuracy of the surface deformation information. 

PS-InSAR is a commonly used method with the time series InSAR technique, which can obtain high-precision surface deformation information. PS-InSAR is suitable for urban areas or areas with stable interferometric conditions and radiation, but its applicability is limited by the small number of PS points and their sparse distribution under natural surface conditions.

The selected *N* images are formed into a data set on the time series, and the *i*th image of this data set is denoted as $S_{i}(i=1,2,\cdot \cdot \cdot ,N)$. The interferogram of the conjugate image pair *i* and *j* is recorded as $I^{i,j}=s_{i}s_{j}^{*}$. The phase difference between the target *p* and the reference point *p*_0_ are denoted as Equations (1) and (2):

(1) $$\Delta \phi_{H,p,p_{0}}^{i,j}=\frac{4\pi }{\lambda }\frac{1}{R\sin\theta }\Delta h_{p,p_{0}}B_{n}^{i,j}$$

(2) $$\Delta \phi_{D,p,p_{0}}^{i,j}=\frac{4\pi }{\lambda }\Delta v_{p,p_{0}}B_{t}^{i,j}$$

In Equation (1), *λ* is the wavelength; *R* is the distance between the detection point and the radar imaging point; *θ* is the angle of incidence indicating the SAR image; $\Delta \;h_{p,p_{0}}$ and $\Delta v_{p,p_{0}}$ are the elevation and deformation rate differences between the two targets, respectively; and $B_{n}^{i,j}$ and $B_{t}^{i,j}$ denote the spatio-temporal baselines of SAR images *i* and *j* in the vertical direction, respectively.

The rate of deformation of the ground monitoring point in the vertical direction can be expressed by maximising the temporal coherence $\xi_{p}$:

(3) $$(\Delta \hat{h_{p}},\Delta \hat{v_{p}})=\arg\left\{\max\left(\left|\xi_{p}\right|\right)\right\}$$

Among these,

(4) $$\xi_{p}=\frac{1}{M}\sum_{i,j}e^{j(\Delta \phi_{p}^{i,j}-\Delta \phi_{H,p}^{i,j}-\Delta \phi_{D,p}^{i,j})}$$

In Equation (4), *M* denotes the number of interferograms; $\Delta \phi_{p}^{i,j}$ denotes the interferometric phase of the *i*th and *j*th images after de-flattening effect and removal of topographic residuals; $\Delta \phi_{H,p}^{i,j}$ is the partial phase associated with the elevation; and $\Delta \phi_{D,p}^{i,j}$ represents the associated phase due to surface deformation. After interferometric processing of the image data, the deformation result in the line-of-sight direction can be expressed in two parts:=, linear and nonlinear deformation, as Equation (5).

(5) $$\phi_{D,p}^{i,j}=\phi_{linear}^{i,j}+\phi_{non-linear}^{i,j}=\frac{4\pi }{\lambda }(\Delta v_{p}B_{t}^{i,j}+Defo_{non-linear})$$

The temporal coherence is related to the divergence of the phase residuals, as shown by Equation (6):

(6) $$\hat{\xi_{p}}=e^{-\frac{\delta_{\phi }^{2}}{2}}$$

The deformation rate variance and elevation DEM variance of the target and reference points, i.e., $\delta_{\Delta v}^{2}$ and $\delta_{\Delta h}^{2}$with respect to the phase divergence, interferogram geometry with respect to the temporal baseline divergence, and the *M*-amplitude interferograms, can be derived as Equation (7):

(7) $$\left\{ \begin{array}{l} \delta_{\Delta h}^{2}\approx {\left(\frac{\lambda R\sin\theta }{4\pi }\right)}^{2}\frac{\delta_{\phi }^{2}}{M\delta_{Bn}^{2}}\\ \delta_{\Delta v}^{2}\approx {\left(\frac{\lambda }{4\pi }\right)}^{2}\frac{\delta_{\phi }^{2}}{M\delta_{Bt}^{2}} \end{array}\right.$$

The PS-InSAR method identifies individual, stable, permanent scatterers via the correlation of pixel values in the acquired SAR image in phase in space and time, and is not affected by temporal or spatial decoherence.

#### 2.3.2. DS-InSAR Method

While the PS-InSAR technique relies on stable scattering to measure surface deformation, the key advantage of DS-InSAR is its ability to measure surface deformation in areas (e.g., agricultural fields or vegetated areas) where there are no persistent, stable point targets (e.g., buildings or rocks).

The key to the identification of DS points is to select all pixel points within the estimation window that are statistically homogeneous to the desired pixel at a given significance level. The FaSHPS (fast SHP selection) algorithm was used to compute the SHP used to evaluate amplitude data with amplitude from the same probability density function (p.d.f).

The core idea of the FaSHPS (fast SHP selection) algorithm [39] is to transform the hypothesis testing problem into confidence interval estimation, and to judge whether the image metapoints are homogeneous or not according to the similarity between the image metapoints data and the reference point in the time series.

From the central limit theorem, the greater the number of SAR images on the time series, the closer the mean amplitude $\bar{A}(M)$ will be to a Gaussian distribution, in which case, the interval estimate of $\bar{A}(M)$ can be expressed as Equation (8) [39]:

(8) $$P\left\{\mu (M)-z_{1-\alpha /2}\cdot \sqrt{Var\left(A(M)/N\right)}<\bar{A}(M)<\mu (M)+z_{1-\alpha /2}\cdot \sqrt{Var\left(A(M)/N\right)}\right\}=1-\alpha$$

where *P*{·} denotes the probability, $z_{1-\alpha /2}$ denotes the 1-α/2 quantile in the standard normal distribution, *μ*(*M*) is the expectation of the image element *M*, *Var*(*A*(*M*)) denotes the true variance of the amplitude of the image element *M* over the time series, and *N* denotes the number of SAR images.

In the homogeneous region, it can be assumed that the amplitude of the SAR image obeys the Rayleigh distribution [40]; then, the coefficient of variation CV can be calculated using Equation (9):

(9) $$\mathrm{CV}=\sqrt{Var(\cdot )}/E(\cdot )=\sqrt{4/\pi -1}\approx 0.52$$

where *E*(·) denotes the expectation. Assuming that the scattering characteristics of the image element are essentially stable over the time range of observation, Equation (8) can be converted to:

(10) $$P\left\{\mu (M)-z_{1-\alpha /2}\cdot \frac{0.52\cdot \mu (M)}{\sqrt{N}}<\bar{A}(M)<\mu (M)+z_{1-\alpha /2}\cdot \frac{0.52\cdot \mu (M)}{\sqrt{N}}\right\}=1-\alpha$$

Taking $\bar{A}(M)$as the truth value of the mean amplitude of the image element *S* in the time dimension, the confidence interval of the mean amplitude can be found according to Equation (10). The mean amplitude of the neighbouring image elements on the time series can be calculated, and if it is within the confidence interval of the mean amplitude of the reference image element, the two are considered homogeneous.

In distributed target image elements, features should exhibit consistent scattering characteristics. Due to the interference of noise and spatio-temporal incoherence, the signal vector of each image element contains multiple scattering signal types in time-series SAR image interferograms, resulting in poor phase stability of distributed target image elements. Optimisation of image element phases can have the effect of reducing the phase noise of interferometric image elements [41]. The eigenvalues associated with the image element coherence matrix characterise different backscattering properties, and by performing an eigenvalue decomposition of the coherence matrix [42], the largest eigenvalue and its corresponding eigenvector can be determined. The image element scattering characteristic corresponding to this eigenvector is considered to be stable and is used as the optimised phase.

Assuming that there are *N_P_* image elements with similar backscattering properties in the uniform region *Ω*, their coherence matrix *Y* can be expressed as Equation (11):

(11) $$Y=E\left[xx^{H}\right]\approx \frac{1}{N_{P}}\sum_{x=\Omega }xx^{H}$$

where *x* = [*x_1_*, *x_2_*, …, *x_N_*] is the complex vector of the complex observations of the homogeneous points of the distributed target on the *N*-scene SAR image after normalisation, and (·)^H^ denotes the conjugate transpose of the matrix. The coherence matrix obtained using the above equation is a semi-positive definite Hermitian matrix, which can be obtained by eigenvalue decomposition:

(12) $$Y=B\Lambda B^{-1}=B\Lambda B^{H}=\sum_{i=1}^{N}\lambda_{i}\cdot b_{i}\cdot b_{i}^{H}$$

where Λ is the diagonal matrix of non-negative real eigenvalues *λ_i_*, and *Y* is the orthogonal eigenvector corresponding to different eigenvalues. The larger *λ_i_* is, the more dominant the corresponding feature scattering mechanism becomes, so the eigenvector *μ*_1_ corresponding to the largest eigenvalue *λ_1_* can be taken as the phase corresponding to the main scatterer. From this, the main scatterer signal *Y* signal can be calculated, and the rest is used as the decoherent noise signal *Y* noise. By eliminating the phase component of the decoherent noise signal and retaining the phase component of the main scatterer signal, we can achieve phase optimisation:

(13) $$Y=Y_{signal}+Y_{noise}=\lambda_{1}\cdot \mu_{1}\cdot \mu_{1}^{H}+\sum_{i=2}^{N}\lambda_{i}\cdot \mu_{i}\cdot \mu_{i}^{H}$$

Once the phase optimisation is complete, the quality of the phase optimisation also needs to be assessed. The goodness of fit is calculated by fitting the difference between the interferometric phases before and after optimisation [25]:

(14) γDS=2N(N−1)Re∑n=1N∑o=n+1Nexpj(φno−(φo−φn))

where $\gamma_{DS}$ is the goodness of fit, which can also be viewed as the temporal coherence of the distributed target; $\varphi_{no}$ denotes the interfering phase of the two SAR images before optimisation; $\varphi_{n}$ and $\varphi_{o}$ denote the phase of the two SAR images after optimisation, respectively. 

#### 2.3.3. SBAS-InSAR Method

Differential interferometry is performed separately for each set of image pairs within group *K*. Assuming that the final number of eligible differential interferograms that can be acquired is *M*, then:

(15) $$\frac{N+1}{2}\ll M\ll N\left(\frac{N+1}{2}\right)$$

Assuming that the main and auxiliary images acquired at the moments *t_A_* and *t_B_*, respectively, are multiplied by the complex conjugate phase to obtain the *i*-scene differential interferogram, if *x* denotes the azimuthal coordinate of the image element on the map and *r* denotes the corresponding distance coordinate, the interferometric phase of any image element on the differential interferogram can be expressed by Equation (16), as follows:

(16) $$\begin{array}{c} \delta \phi_{k}(x,r)=\phi (t_{B},x,r)-\phi (t_{A},x,r)\approx \frac{4\pi }{\lambda }\left[d(t_{B},x,r)-d(t_{A},x,r)\right]+\frac{4\pi }{\lambda }*\frac{B_{⊥j}\Delta z}{r\sin s\theta }+\\ {}[\phi_{atm}(t_{B},x,r)-\phi_{atm}(t_{A},x,r)]+\Delta n_{j}\forall j=1,\cdots \;,M \end{array}$$

From the above equation, λ is the SAR wavelength; $d(t_{B},x,r)$ and $d(t_{A},x,r)$ are the accumulated deformations in the line-of-sight direction of the image element at *t_B_* and *t_A_* with respect to the original moment *t*_0_, respectively; for the time being, $d(t_{0},x,r)\equiv 0$; $\frac{4\pi }{\lambda }*\frac{B_{⊥j}\Delta z}{r\sin s\theta }$ is the residual terrain phase, where $B_{⊥j}$ denotes the vertical baseline distance and Δ*z* denotes the terrain error; *θ* denotes the incidence angle; $\phi_{atm}(t_{B},x,r)-\phi_{atm}(t_{A},x,r)$ represents the atmospheric delay phase at the time of imaging; and Δ*n_j_* denotes the noise phase.

Afterwards, all the obtained interferograms are processed with phase filtering and phase de-entanglement using points with known deformation or points in the stable region of the surface, and the coherence coefficient information can be used to select the points with higher coherence as the points of the GCPs. The following is an example of an arbitrary image element to illustrate the subsequent processing:

Suppose that $\phi^{T}$ represents a vector consisting of the phases of some highly coherent image element at time $\left(t_{1},\cdot \cdot \cdot ,t_{N}\right)$, denoted as Equation (17):

(17) $$\phi^{T}=\left[\Phi (t_{1}),\cdot \cdot \cdot ,\Phi (t_{N})\right]$$

$\delta \Phi^{T}$ denotes the phase vector of each interferogram; then, it can be expressed as Equation (18):

(18) $$\delta \Phi^{T}=\delta \Phi_{1}\ldots \delta \Phi_{M}$$

Assuming that the primary and secondary images are arranged in time series as $IE=[IE_{1}\cdots IE_{M}]$ and $IS=[IS_{1}\cdots IS_{M}]$, and ${\mathrm{IE}}_{k}>IS_{k}$, ∀k=1,⋯ ,M, based on the time series, the corresponding phase of the *j*th view image is:

(19) $$\delta \phi_{j}=\phi \left(t_{IE_{j}}\right)-\phi \left(t_{IS_{j}}\right)\forall k=1,\cdots \;,M$$

In the above equation, if the *A* matrix is expressed as *M* × *N*, the above equation can be written as Equation (20):

(20) Aϕ=δϕ

The rows in matrix *A* in the above equation correspond to the coherence coefficients of each solution entanglement. Using *M* equations to solve the corresponding phases of the *N*-scene radar image, when the time is M≫N and the rank of the matrix *A* is *N*, it can be solved using the least-squares method, and the solution values are indicated by Equation (21):

(21) $$\hat{\phi }={\left(A^{T}A\right)}^{-1}A^{T}\delta \phi$$

In practice, due to the influence of temporal and spatial baselines, the paired image pairs will not belong to only a certain small set of baselines. The SVD method is used in data processing. However, the use of the SVD method will cause the deformation accumulation to become discontinuous in time, violating its physical law, so the average rate of the phase between adjacent times can also be solved as an unknown quantity. The process is shown by Equation (22):

(22) $$v^{T}=\left[\begin{array}{c} v_{1}=\frac{\phi_{1}}{t_{1}-t_{0}}\cdots v_{N}=\frac{\phi_{N}-\phi_{N-1}}{t_{N}-t_{N-1}} \end{array}\right]$$

Equation (21) can be replaced with Equation (23):

(23) $$\sum_{k=lS_{k+1}}^{IE_{k}}(t_{i}-t_{i-1})v_{k}=\delta \phi_{k}$$

Finally, the temporal low-pass deformation component of the surface deformation is calculated using the least squares method according to the following equation. The terrain error and the results of the surface deformation processed by temporal low-pass filtering, as well as the terrain error, are obtained using the least squares method according to Equation (24):

(24) $$[BM,c]P_{c}=\delta \phi$$

where *B* is an *M* × *N* matrix of coefficients, typically with C for elements (*j*, *k*) when $IS_{j+1}\leq k\leq IS_{j}$, ∀j=1,⋅⋅⋅,M, and $B(j,k)=t_{k+1}-t_{k}$ roots for the rest of the cases. *M* denotes the vector portion of the rate *v*; *M* denotes the vector portion of the rate *v*; $C^{T}=\left[\left(\frac{4\pi }{\lambda }\right)\left(\frac{B_{⊥1}}{r\sin\theta }\right)...\left(\frac{4\pi }{\lambda }\right)\left(\frac{B_{⊥M}}{r\sin\theta }\right)\right]$; $P_{C}^{T}=[P^{T},\Delta z]$; *P* denotes the temporal low-pass component of the phase after de-entanglement; the three letters in $P^{T}=[\bar{v},\bar{a},\Delta \bar{a}]$ stand for mean velocity, mean acceleration, and mean variance acceleration; and Δ*z* denotes topographic error.

The InSAR LOS values are, in fact, a projection of the vertical values in the LOS direction, with the following conversion relationship (Equation (25)):

(25) $$r=\frac{d}{\cos\theta }$$

where *r* is the vertical direction displacement value, *d* is the line-of-sight direction displacement value, and *θ* is the angle of incidence. We can convert the InSAR value to the vertical direction value.

SBAS-InSAR is a nonlinear sedimentation model, and PS-InSAR and DS-InSAR are linear sedimentation models. The sedimentation processes described by of the two are not exactly the same, and there exists local consistency or local inconsistency. However, PS-InSAR, DS-InSAR, and SBAS-InSAR have complementary advantages. In this study, a PS-DS four-threshold (coherence coefficient threshold, FaSHPS adaptive threshold, amplitude divergence index threshold, and deformation velocity interval) method combining PS and DS points is proposed as a SBAS-PS-DS-InSAR method for selecting PS and DS points as the GCPs for orbital refinement and re-flattening.

#### 2.3.4. SBAS-PS-DS-InSAR Method

**Figure 2:**
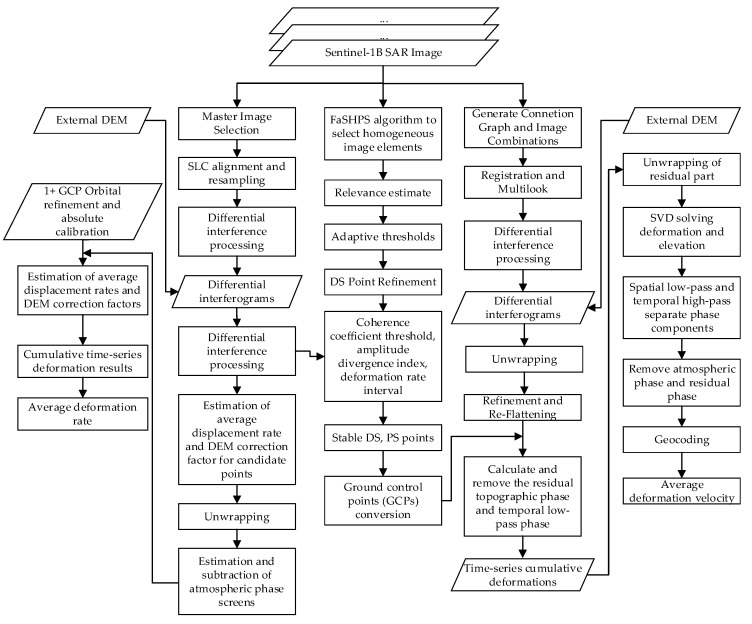
Technical flow chart.

The InSAR-based surface deformation monitoring study of Tongliao urban area proposed herein is mainly divided into five steps, and the technical flow is shown in Figure 2 below:

(1) Data pre-processing. Sentinel-1B satellite SLC image data, DEM data, and precision orbit data of the study area were downloaded. Data clipping and baseline data estimates were made according to the extent and characteristics of the study area.
(2) PS-InSAR and DS-InSAR processing were performed on the image data processed in step (1) to identify PS points and DS points waiting for screening.
(3) PS-DS four-threshold method (coherence coefficient threshold, FaSHPS adaptive threshold, amplitude deviation index threshold, and deformation velocity interval) processing was used to obtain stable and eligible DS and PS points as GCPs points.
(4) SBAS-PS-DS-InSAR processing. The GCPs selected using the PS-DS four-threshold method were used in the orbit refinement and re-flattening steps of the SBAS-InSAR processing, and then the time series deformation results were obtained by deformation inversion.
(5) Comparison verification and analysis. The deformation results of SBAS-InSAR and SBAS-PS-DS-InSAR monitoring were verified and analysed in comparison with the GPS monitoring results to provide a reference for urban surface deformation disaster prevention and municipal planning.

#### 2.3.5. Ground Control Point Screening

Ground control points (GCPs) are used for track refinement and re-flattening to estimate and remove residual phases and phase ramps remaining after unwinding, as well as to improve the accuracy of deformation monitoring [43].

The selection principles of GCPs need to satisfy the following points [34]:

- High coherence, good de-entanglement results, and stable regions;
- Areas without deformation fringes and away from deformation;
- Regions without residual topographic streaks;
- No phase jumps, as they cannot be located in isolated phases;
- Uniformly distributed in the image.

The manual selection of GCPs can cause large errors. Therefore, this study proposes a four-threshold method based on PS and DS to identify GCPs. Firstly, the coherence coefficient thresholding method [44] is used to identify the PS point targets with high coherence, and the FaSHPS method is used to adaptively threshold the DS point targets [45]; then, the amplitude deviation value [39] is set to further screen the more stable PS and DS points as the targets; and, finally, a deformation rate interval is set to carry out the final PS and DS point selection.

(1) Coherence factor method

The distribution interval of the coherence coefficient values is [0,1], with 0 indicating complete incoherence and 1 indicating complete coherence. According to the characteristics of PS points, the corresponding coherence coefficient in the region of good coherence is also relatively high [40], so the coherence coefficient can be used as the judgement threshold for PS and DS point selection, which is expressed as Equation (26) [33]:

(26) $$\gamma =\frac{|\sum_{i=1}^{m}\sum_{j=1}^{n}M(i,j)\cdot S^{*}(i,j)|}{\sqrt{\sum_{i=1}^{m}\sum_{j=1}^{n}|M(i,j){|}^{2}\cdot \sum_{i=1}^{m}\sum_{j=1}^{n}|S(i,j){|}^{2}}}$$

where *M* (*i, j*) is the primary image, *S* (*i, j*) is the secondary image, and * is the conjugate multiplication. The coherence coefficient is calculated for each pixel on the time series. Assuming that there are *M* + *1* SAR images, *M* interferograms can be formed, and each image element forms a coherence coefficient number sequence $\gamma_{m}=(m=1,2,\cdot \cdot \cdot ,M)$ [45].

(27) $$\bar{\gamma }=\frac{1}{M}\sum_{i=1}^{N}\gamma_{i}$$

A coherence threshold is set, and when the value of an image element is greater than this threshold, it is determined to be a valid PS point [46].

(2) FaSHPS adaptive thresholding method

Assuming a true coherence threshold of $\varepsilon_{T}^{\mathrm{inint}}$ (points above $\varepsilon_{T}^{\mathrm{inint}}$ are DS points), the adaptive threshold is:

(28) $$\varepsilon_{T}=\varepsilon_{T}^{\mathrm{inint}}+k\sigma_{\varepsilon }$$

where *k* is a real constant such that *k* = 1, and *σ_ε_* denotes the lower bound standard deviation of Gramer–Rao for unbiased coherence:

(29) $$\sigma_{\varepsilon }=\frac{1-\varepsilon_{T}^{\mathrm{inint}}}{\sqrt{2R}}$$

*R* denotes the number of homogeneous points within the estimation window.

For any to-be-estimated point in space, all coherence samples in time are compared with $\varepsilon_{T}$, and if the number of samples with unbiased estimates of coherence greater than or equal to the adaptive threshold $\varepsilon_{T}$ is greater than a given acceptable percentage of images (here set to 85%), they are considered DS candidates [39].

(3) Amplitude dispersion index method

In 2001, Ferretti et al. proposed dispersion of amplitude (DA), which is based on the definition of PS. When the image element is satisfied with the presence of the main scatterer, its phase is mainly determined by the phase of the main scatterer, which is less affected by the noise, etc., and the phase standard deviation and amplitude exist in the following Equation (30) [46]:

(30) $$\sigma_{\varphi }\approx \frac{\sigma_{A}}{m_{A}}≜D_{A}$$

where $\sigma_{\varphi }$ is the standard deviation of the phase; $\sigma_{A}$ is the standard deviation of the amplitude *A*; $m_{A}$ is the mean value of the amplitude of the *N* SAR images in the time dimension; and $D_{A}$ is the divergence index. When $D_{A}$ is below a certain threshold, the PS points and DS points are selected.

In this study, stable PS and DS points are selected via PS-InSAR and DS-InSAR processing and by using the coherence coefficient, FaSHPS adaptive threshold method, amplitude deviation index, and deformation rate. Firstly, the high coherence pixels are selected as PS points using the coherence coefficient method, the coherence threshold is set to 0.95, and the pixels larger than this threshold are selected as PS points. The pixels whose coherence meets the adaptive threshold are selected as DS points using the FaSHPS adaptive threshold method, and the true coherence threshold is set to 0.56. Secondly, the stable PS and DS points are selected via the amplitude divergence method, the amplitude divergence index threshold is set to 0.56, and the stable PS points are selected using the amplitude divergence index threshold. The amplitude divergence index threshold is set to 0.25, and PS and DS points smaller than this threshold are retained. Finally, the deformation rate is used to determine the final selected PS and DS points, and the deformation rate interval is set to [−0.3 mm/a, 0.3 mm/a]. According to the above four-threshold method, a total of 23 qualified PS and DS points are selected as the GCPs, which are used for track refinement and re-flattening so as to obtain more accurate results of the surface deformation monitoring.

#### 2.3.6. SBAS-PS-DS-InSAR Processing

In the SBAS-PS-DS-InSAR method, one image is selected as the super-master image and the rest of the images are used as slave images. The connectivity map is determined using a time–space baseline threshold. The deformation monitoring accuracy of this method increases as the spatial baseline decreases. The temporal–spatial baseline thresholds are set to 2% and 120 d of the maximum critical baseline, respectively, and the image interference pairs are generated according to the principle of small baseline set. Next, a series of processing steps are carried out, including the elimination of flat and terrain phases, generation of differential interferograms, filtering of interferometric phase maps, and phase untangling. By employing the four-threshold method of PS-DS, the PS points are automatically selected and the DS points are converted into GCPs, which are subsequently used in the track refinement and re-flattening steps. Once the above steps are completed, the cumulative time-series deformation results of the study research region can be obtained, and then the average deformation rate of the study region is obtained after the steps involving SVD and residual atmospheric phase removal.

### 2.4. GPS Measurements

In this study, GPS measurements were carried out using GPS-RTK technology [47], which is a combination of GPS measurement technology and data transmission technology. Its application in measurement is based on carrier phase measurement, which is capable of performing real-time differential GPS measurements, where the carrier phase collected by the reference station is sent to the user receiver for differential resolution of the coordinates. The GPS-RTK measurement system generally consists of the following three parts: GPS receiver equipment, data transmission equipment, and a software system [48]. It has the advantages of high measurement accuracy, real-time mapping function, and strong adaptability in the process of practical application [49].

## 3. Results and Analysis

In this study, 45 views of Sentinel-1B SAR imagery data from 16 February 2019 to 14 December 2021 in the urban area of Horqin District, Tongliao City, Inner Mongolia Autonomous Region were processed using the PS-InSAR, SBAS-InSAR, and SBAS-PS-DS-InSAR methods.

**Figure 3:**
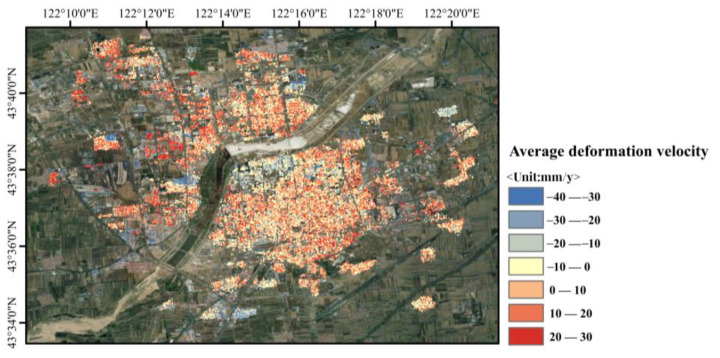
Mean deformation rate of urban area in Horqin District, Tongliao City, monitored using the PS–InSAR technique.

**Figure 4:**
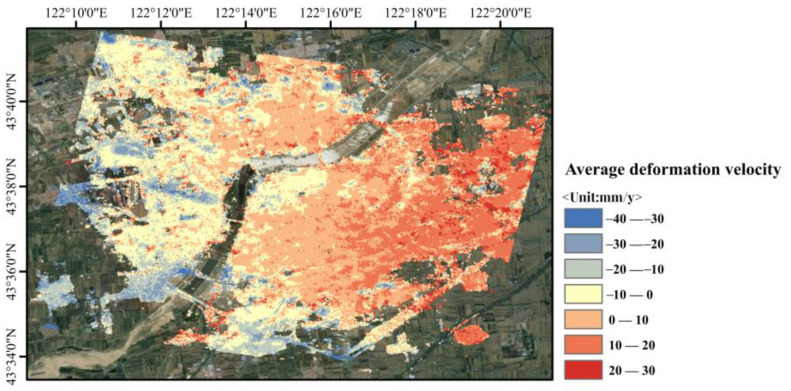
Mean deformation rate of urban area in Horqin District of Tongliao city, monitored using SBAS-InSAR technology.

**Figure 5:**
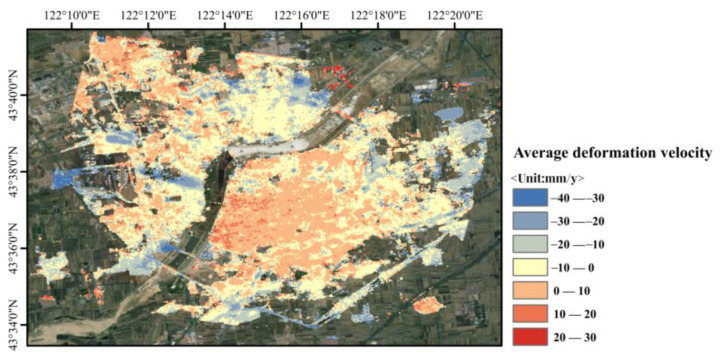
Mean deformation rate of urban area in Horqin District of Tongliao City, monitored using SBAS-PS-DS-InSAR technology.

**Figure 6:**
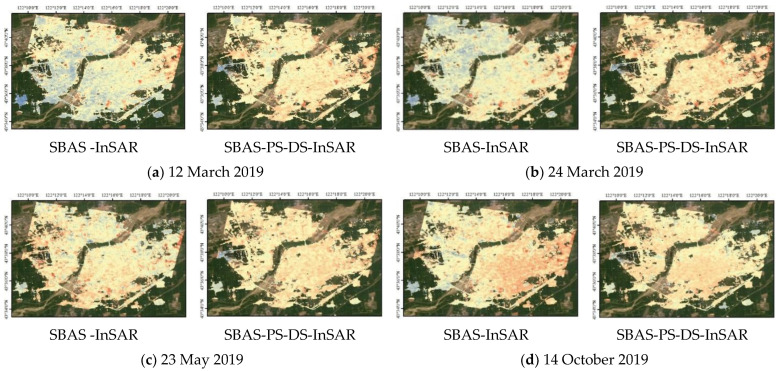

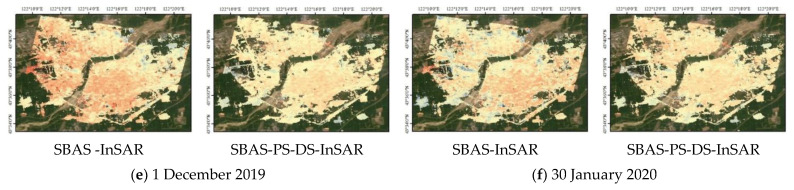

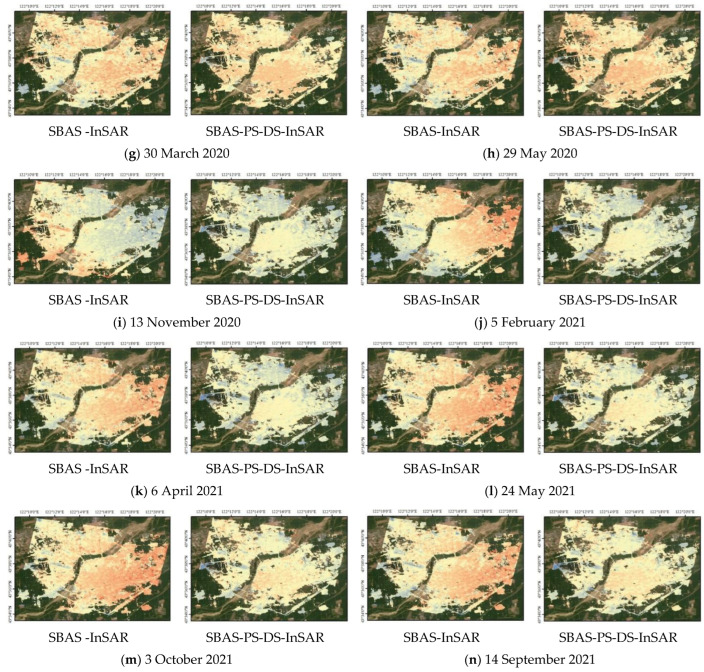
Comparison of temporal deformation results for simultaneous monitoring using the SBAS-InSAR and SBAS-PS-DS-InSAR methods.

In this study, two techniques, SBAS-InSAR and SBAS-PS-DS-InSAR, were combined and compared in order to process the 45-view Sentinel-1B SAR imagery data for the urban area of Horqin District, Tongliao City, Inner Mongolia Autonomous Region, for the period of 16 February 2019 to 14 December 2021, as well as to validate and analyse the monitored deformation results. For the processing of InSAR technology, the selection of ground control points for orbit refinement and re-flattening is a necessary step. This helps to determine the precise position and elevation information of the satellite orbit and topography, thus improving the accuracy of the measurements and counteracting the influence of non-surface deformation factors. It also improves the quality of the surface deformation solution using the traditional InSAR method, so as to increase the accuracy of the surface deformation monitoring and to restore the surface deformation process accurately. The stable PS and DS points selected using the PS-DS four-threshold method basically met the requirements for the selection of ground control points, which are used for orbit refinement and re-flattening. The average deformation rates in line-of-sight of urban areas in Horqin District of Tongliao City, which were monitored using PS-InSAR, SBAS-InSAR, and SBAS-PS-DS-InSAR, are shown in Figure 3, Figure 4 and Figure 5, and a comparison of temporal deformation results from the same period of time monitoring between the SBAS-InSAR and SBAS-PS-DS-InSAR methods is shown in Figure 6.

Comparing Figure 3 with Figure 4 and Figure 5, it can be seen that, with PS-InSAR, it is difficult to monitor deformation information in less coherent areas compared to SBAS-InSAR and SBAS-PS-DS-InSAR. In addition, according to the results of the deformation, the settlement areas were located in less coherent areas, so we did not compare the results of PS-InSAR for the purpose of analysing and discussing the results of PS-InSAR. From the surface deformation results shown in Figure 4 and Figure 5, it can be seen that from 16 February 2019 to 14 December 2021, both the SBAS-InSAR and SBAS-PS-DS-InSAR methods detected a large area of uplift and a few areas of subsidence in the study area. We observed the monitoring results of both SBAS-InSAR and SBAS-PS-DS-InSAR methods, both of which detected distinct subsidence funnels extending around the centre of the subsidence funnel. As shown in the temporal deformation process in Figure 6, we found that, along with the lifting of the large area, the subsidence in some areas also accumulated gradually. The scope of subsidence spread outward around the centre of the subsidence funnel, and the surface subsidence showed a multicentre aggregation. The two methods of SBAS-InSAR and SBAS-PS-DS-InSAR revealed that, within the study area, Ershueshu, Qianjin Village, No. 2 Gacha, Zhanlu Village, San Yitang Village, Yanjiaweizi Village, both sides of Hongguang Bridge of Xiliao River, both sides of Shengli Road Bridge, the intersection of Aoxi Section Highway and Dazheng Line Railway, Xinglong Village, Zherimim Station, the intersection of Jitong Line Railway and Tonghuo Line Railway, Tongliao Forest Park, Tongdetian Village, and other areas with poor coherence experienced more serious subsidence. Dadequan Village, Jinbotun, Gaojiayao Village, New Century Bridge, Zherimim Bridge, Qinghe Street and Chuangye Avenue, Tongliao Municipal Government Square, Kulun Road and Liuyin Road, Hanshan Street and National Highway 111, and Fuli City all experienced more serious uplift. For this study area, the maximum cumulative subsidence monitored by both the SBAS-InSAR and SBAS-PS-DS-InSAR methods were −90.78 mm and −83.68 mm, respectively, the maximum cumulative uplift values were 74.94 mm and 97.56 mm, respectively; and the annual average maximum subsidence rates were −35.38 mm/y and −30.38 mm/y, respectively. The annual average maximum uplift rates were 25.27 mm/y and 27.92 mm/y, respectively. Overall, the two methods used to monitor the location, scope, distribution, and average deformation rate of surface subsidence in urban areas showed similarity. However, specific analyses revealed that the cumulative subsidence monitored by SBAS-InSAR was larger than that by SBAS-PS-DS-InSAR, while the cumulative uplift monitored by SBAS-InSAR was smaller than that by SBAS-PS-DS-InSAR in the same time period.

**Figure 7:**
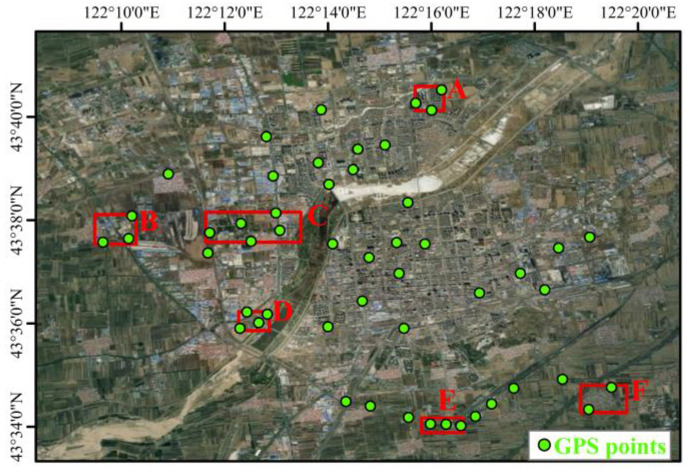
Locations of deformation areas A–F.

Six deformed regions (A–F) were selected for the purpose of analysing and comparing their time series, and the locations of deformed regions A-F are shown in Figure 7. Areas A, B, C, D, and E were located in large subsidence areas, and area F was in an area of intensive uplift. Considering that some of the SBAS-PS-DS-InSAR monitoring results and the locations of the GPS monitoring points were missing, the six areas were taken as the focus of the study of deformation in the urban area so as to reflect the deformation in the urban area of Tongliao City, Horqin District. The InSAR monitoring results were as follows: Area A was located at the intersection of Qinghe Street and Wulanhua Road, where a subsidence funnel occurred, and the annual average maximum subsidence rate of the subsidence centre was −22.65 mm/y. Area B had a larger subsidence range, but did not have a subsidence funnel. The annual average maximum subsidence rate was −27.69 mm/y, and the subsidence range included the two tree villages as well as the Tongliao inland port and the Eurasia Vocational High School. In Area C, an east–west shuttle-shaped subsidence funnel was formed, with an annual average maximum subsidence rate of −27.79 mm/y at the centre of subsidence, and the scope of subsidence included Zhelimu Station and the nearby vegetation cover area. Area D was located on the west bank of Hongguang Bridge of Xiliao River, with an average maximum subsidence rate of −24.62 mm/y at the centre of subsidence. The area around the subsidence funnel was also affected by subsidence, forming a large area of subsidence. E area was located in the western section of the road of Aoxi and Dazheng Line Railway Interchange, and the average annual maximum subsidence rate of the subsidence centre was −16.36 mm/y. Area F was located in Dadequan Village, which showed an overall uplift trend, with an average maximum uplift rate of 6.06 mm/y. 

**Figure 8:**
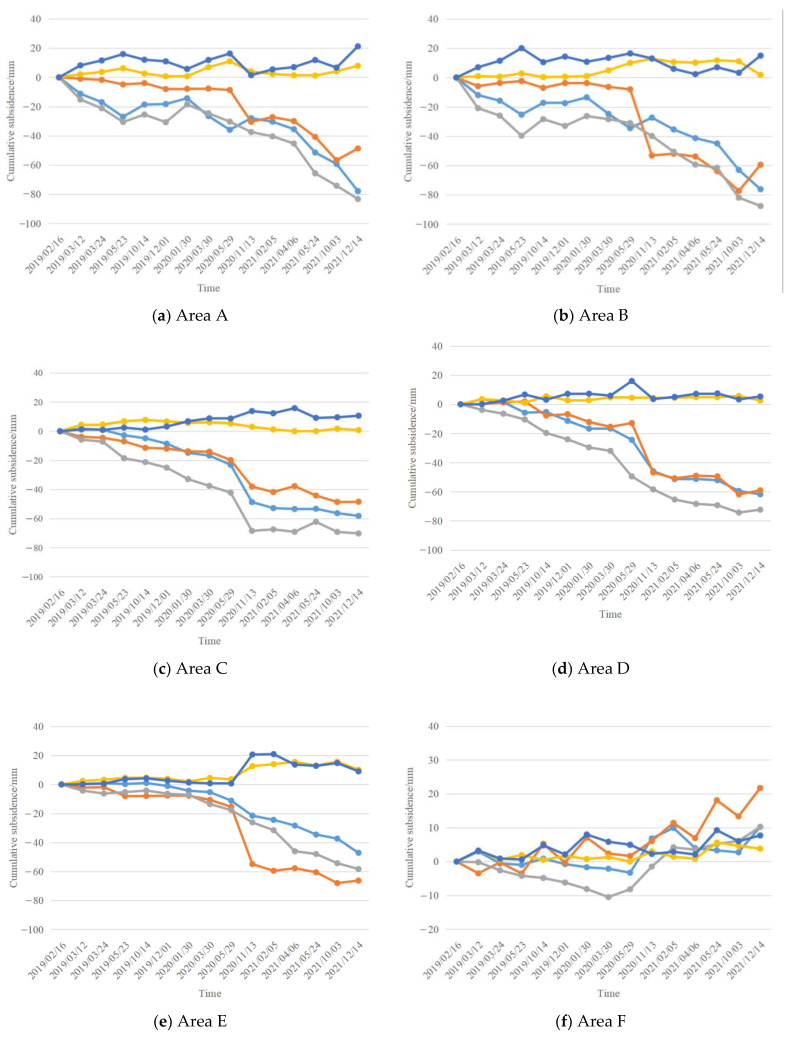

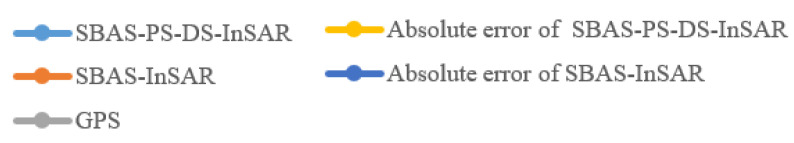
Time-series sedimentation results for areas A-F, monitored using SBAS-InSAR and SBAS-PS-DS-InSAR.

Since the GPS monitoring results denote vertical deformation, the geometrical relationship between the InSAR vertical and line-of-sight directions was used to convert the line-of-sight deformation results to vertical deformation results for easy comparison. The following section compares the InSAR vertical deformation results with the GPS monitoring results. In the comparison experiment, the GPS measurements were averaged over the area marked in red in Figure 8 according to the latitude and longitude of the GPS measurement points, and the same was carried out for the corresponding InSAR point measurements, comparing the average values of the deformations monitored by the three methods over the area. The averaged values were based on the number of GPS points falling within the red-marked area. The time series subsidence results of areas A–F, monitored by the SBAS-InSAR method and the SBAS-PS-DS-InSAR method, are shown in Figure 8. In the study area, the SBAS-InSAR and SBAS-PS-DS-InSAR methods were basically consistent with the GPS detection results, and the deformation curves of the three methods showed similar trends. However, the deformation curves monitored by the SBAS-InSAR method were steeper, with a sudden drop between 29 May 2020 and 13 November 2020, while the curves monitored by the SBAS-PS-DS-InSAR method were smoother relative to them. Taking Figure 8c as an example, the time-series deformation map of the region shows the following information: From 16 February 2019 to 29 May 2020, the amount of subsidence was small, and the deformation was smooth but with a slight downward trend; more significant subsidence occurred between 29 May 2020 and 6 April 2021. From 6 April 2021 to 14 December 2021, although there was still subsidence occurring, the subsidence trend became more gradual. In short, the area experienced two different phases of subsidence during the observed time period, with slower deformation trends in the early and late periods and a significantly larger change in the middle period. As can be seen from Figure 8c, the time-series deformation basically showed a subsidence trend. The time-series subsidence trend was basically smaller and slower in the early and late stages, with a larger subsidence inflection point in the middle stage, and the inflection point occurred at basically the same time. Taking Figure 8f as an example, the time-series deformation map of the region shows the following information: Between 16 February 2019 and 29 May 2020, it lifted and then subsided, but the overall deformation was relatively small. A more pronounced lifting occurred between 29 May 2020 and 5 February 2021, followed by a sudden subsidence, but after 3 November 2021, it continued to lift. In short, the region experienced three uplift–settlement phases during the observed time period, with minor subsidence and a general uplifting trend. This can be seen in Figure 8c: The time-series deformation basically shows an uplifting trend, with smaller subsidence and recurring subsidence and uplift.

In Figure 8, areas (A–E) show the subsidence areas, while area (F) shows the uplift areas. It can be observed from Figure 8a–f that the time series subsidence trends obtained from the SBAS-PS-DS-InSAR method were more consistent with the GPS measured data. In this study, the correlation between the SBAS-InSAR method and the SBAS-PS-DS-InSAR method for the six subsidence areas and the GPS monitoring results were compared. The strength of the linear relationship between the two monitoring results and the GPS data was assessed using the Pearson correlation coefficient, with higher correlation coefficients (values closer to 1) indicating greater consistency in deformation trends. Detailed comparisons and validation analyses of the SBAS-InSAR and SBAS-PS-DS-InSAR methods with the GPS monitoring results were carried out by calculating the Pearson correlation coefficients, the MAE, and the RMSE, along with other statistical metrics. The related comparison results are summarised in Table 3.

## 4. Discussion

The two monitoring methods, SBAS-InSAR and SBAS-PS-DS-InSAR, showed variability in the monitoring results. Through the comprehensive validation analysis in Table 3, we can see that, combined with the Pearson correlation coefficient, both InSAR techniques showed good correlation with the GPS monitoring results, which implies that the ground deformation trends monitored by both of them were roughly the same. The comparison of the data in Table 3 reveals that the Pearson correlation coefficient of the time-series subsidence results obtained using the SBAS-PS-DS-InSAR method was closer to 1 than that of the GPS monitoring data, which indicates that the SBAS-PS-DS-InSAR method was better in terms of the consistency and accuracy of the monitoring results. In addition, it can be seen from Table 3 and Figure 8 that the time series deformation results measured by the SBAS-PS-DS-InSAR method were reduced in absolute error, MAE, and RMSE compared to the SBAS-InSAR method, and the advantages were more significant, especially in the areas with smaller deformation variables. The surface deformation errors monitored by both InSAR techniques were within acceptable limits, where the monitoring results of the SBAS-PS-DS-InSAR method showed better performances relative to the traditional SBAS-InSAR method. This verifies the validity and reliability of the SBAS-PS-DS-InSAR method.

Overall, the SBAS-PS-DS-InSAR method retains the monitoring characteristics of the traditional SBAS-InSAR method. It is able to accurately locate and monitor the deformation trend of the study area over a large area while basically maintaining the same deformation trend with the monitoring results of the SBAS-InSAR method and the GPS, which shows good validity and credibility. In an area with large deformation, the large deformation gradient and serious incoherence phenomenon will lead to radar image discontinuity. According to the monitoring results, the SBAS-PS-DS-InSAR method is more sensitive and effective for subsidence areas. PS-InSAR and DS-InSAR deformation monitoring is based on the point scale, while SBAS-InSAR and SBAS-PS-DS-InSAR deformation monitoring is based on the surface scale. In this study, the combination of PS and DS improved the shortcomings of these two monitoring tools, which can only be based on the point scale, and incorporated the advantages of SBAS-InSAR, such as large scale and high accuracy. The method solved the limitations of the traditional single InSAR means in terms of application scenarios and computational characteristics, as well as the errors and uncertainties in manual hand-selection of GCPs. It used a combination of DS and PS points to solve the problem of manually-selected GCPs affecting the monitoring results in the process of orbital refining and re-flattening.

## 5. Conclusions

Taking the urban area of Keerqin District, Tongliao City, Inner Mongolia Autonomous Region, China, as the study area, this study used the SBAS-InSAR method and the SBAS-PS-DS-InSAR method to monitor the time-series surface deformation processes in the urban area and compared and analysed these two types of deformation results with the GPS monitoring results. The following conclusions can be drawn:

(1) The SBAS-PS-DS-InSAR method is able to monitor the surface deformation of urban areas effectively in real time, and accurately monitors the location, range, and spatial–temporal distribution of settlements in urban areas. The monitored spatial–temporal deformation is basically the same as that of the GPS method, with basically the same trend.
(2) Comparison between the SBAS-InSAR method and the SBAS-PS-DS-InSAR method reveals that the SBAS-PS-DS-InSAR method can better monitor the surface deformation in urban areas and can effectively obtain the range and distribution of deformation in urban areas. The deformation errors of these two methods are smaller in areas with smaller deformation variables, but the deformation errors of the SBAS-PS-DS-InSAR method are always smaller than those of the SBAS-InSAR method. The Pearson correlation coefficients between the time-series settlement results monitored by the SBAS-PS-DS-InSAR method and the GPS results are always closer than those of the SBAS-InSAR method. The correlation coefficients are always closer than the Pearson correlation coefficients of the SBAS-InSAR method, and the GPS results are always closer to 1.
(3) The surface deformation in urban areas monitored by the SBAS-PS-DS-InSAR method ameliorates the uncertainties and errors that exist when manually selecting the GCPS points manually. The method retains the advantages of PS-InSAR in urban-area applications and the characteristics of DS-InSAR in non-man-made building areas with short vegetation, while also making use of the facet-scale monitoring of SBAS-InSAR to make up the shortcomings of the limited monitoring ranges of PS-InSAR and DS-InSAR.
(4) The SBAS-PS-DS-InSAR method has shown good reliability and accuracy in practical applications, and can effectively monitor the surface deformation of urban areas in time sequences. It provides comprehensive deformation information and references for comprehensive urban deformation management, urban municipal construction planning, and early warning for disasters, while realising effective urban deformation monitoring.

## Figures and Tables

**Table 1:** Main parameters of the Sentinel-1B data used in this study.

Parameter	Value
Flight direction	Descending
Beam mode	IW
Polarisation	VH
Wave band	C
Wavelength/cm	5.6
Number of images	45
Monitored period	16 February 2019–14 December 2021

**Table 2:** Parameters of the Sentinel-1B image.

No	Image Data	Orbit	No	Image Data	Orbit	No	Image Data	Orbit
1	16 February 2019	014977	16	6 January 2020	019702	31	19 December 2020	024777
2	28 February 2019	015152	17	18 January 2020	019877	32	5 February 2021	025477
3	12 March 2019	015327	18	30 January 2020	020052	33	17 February 2021	025652
4	24 March 2019	015502	19	11 February 2020	020052	34	1 March 2021	025827
5	5 April 2019	015677	20	23 February 2020	020402	35	13 March 2021	026002
6	29 April 2019	016027	21	6 March 2020	020577	36	6 April 2021	026352
7	23 May 2019	016377	22	18 March 2020	020752	37	18 April 2021	026527
8	4 June 2019	016552	23	30 March 2020	020927	38	30 April 2021	026702
9	14 October 2019	018477	24	11 April 2020	021102	39	12 May 2021	026877
10	26 October 2019	018652	25	23 April 2020	021277	40	24 May 2021	027052
11	7 November 2019	018827	26	5 May 2020	021452	41	3 October 2021	028977
12	19 November 2019	019002	27	17 May 2020	021627	42	15 October 2021	029152
13	1 December 2019	019177	28	29 May 2020	021802	43	27 October 2021	029327
14	13 December 2019	019352	29	13 November 2020	024252	44	2 December 2021	029852
15	25 December 2019	019527	30	25 November 2020	024427	45	14 December 2021	030027

**Table 3:** Comparison of monitoring results.

Monitoring Methodology	Areas	Pearson Correlation Coefficient	MAE/mm	RMSE/mm
SBAS-InSAR method and SBAS-PS-DS-InSAR method	A	0.87989	11.9061	14.8345
	B	0.82154	14.9627	16.4799
	C	0.98963	6.4014	7.6183
	D	0.98686	3.1196	4.2927
	E	0.95945	14.3706	19.0377
	F	0.66166	4.9479	6.6369
SBAS-InSAR method and GPS method	A	0.93689	17.6760	19.4243
	B	0.86594	16.5225	19.9504
	C	0.96792	16.8147	19.3812
	D	0.95648	14.3293	16.3959
	E	0.95100	8.1740	12.137
	F	0.70984	7.3222	8.6226
SBAS-PS-DS-InSAR method and GPS method	A	0.97034	7.1135	8.3343
	B	0.96693	11.5156	12.7019
	C	0.97767	13.7625	14.8004
	D	0.98202	12.4318	13.6862
	E	0.98711	7.7585	9.1195
	F	0.81091	3.9272	4.7491

## Data Availability

No new data were created in this study. Data sharing is not applicable to this article.
